# New York Letter

**Published:** 1892-07

**Authors:** Wm. L. Russell

**Affiliations:** 151 East 50th St. New York


### *CORRESPONDENCE.*

---

NEW YORK, JUNE 15, 1892.

### PREVENTION OF SYPHILIS.

This subject comes up periodically at some society meeting, and different schemes of prevention are discussed pro and con. The discussions are barren of practical results thus far, but the subject is important enough to demand more notice than it receives.

Dr. L. D. Bulkley read a paper on the syphilis of innocents, at the last meeting of the Section on Hygiene and Public Health. He stated that Fournier had estimated that twenty-four per cent. of syphilitic cases contracted the disease through marital relations, and Ricord had supported the statement, adding that he thought the proportion too low. Fifty per cent. of his own cases had been infected through innocent channels; and among the married, eighty-five per cent. had been infected by the husbands. The effects of the disease were very far-reaching, sterility in the male and female, and death of the fœtus being among the most prominent. In three families which he had observed, one healthy adult alone came of twenty-one births. Scrofula, and race degeneration, and remote degenerative effects still obscure, were to be laid to this disease. Extra-genital lesions were quite common. The virus was usually conveyed by immediate contact, as in kissing, but it was also communicated by indirect contact, as by means of a nursing bottle. This was the case in one instance which he had observed when a husband had infected his wife, a syphilitic infant being born, who in turn infected its nurse and grandparents by means of the nursing bottle.

There were no reliable data from which to judge the prevalence of syphilis in the United States. Sixteen per cent. of all cases on the records of the Marine Hospital Service were classed as syphilitic, and ten per cent. of cases reported from all over the country to the American Dermatological Society were put in the same class.

There were very poor hospital accommodations for these patients in New York; most of them being treated at the dispensaries, and allowed to go abroad to spread the disease. Thus one prostitute was known to have infected three hundred men in ten months. He believed that the disease should be placed in the same category with other contagious diseases, and should be placed under sanitary inspection and control.

Dr. C. W. Allen believed that syphilis had been too long regarded as a purely venereal disease, being regarded by some as a blessing in disguise. More attention should be paid to it as a non-venereal disease, and another name given it, lues perhaps. More adequate instruction should be given to patients, and to physicians. He had known marriage and cohabitation to be permitted by physicians who were ignorant of the contagious nature of the lesions present. Laws already existing, relating to this disease, should be strictly enforced, and new ones should be enacted. It should be made a misdemeanor to wittingly communicate the disease to another. The restraint of hospital patients until the danger of contagion had passed was possible and necessary. In Massachusetts the law required that all inmates of State institutions, if found suffering from syphilis, should be isolated, and if necessary retained beyond the termination of their sentence, until the physician in charge declared them free from contagion.

Dr. E. L. Keyes said that he was opposed to discussion of these subjects in a public manner. The nasty parts of our thoughts as well as of our bodies should be kept from public view. Syphilis was to be regarded in the same light as earthquakes or thunder-showers, things which had always existed and which always would exist. As the disease spread more and more widely, it became less important. The Portuguese were said all to have it, and yet they were a reasonably healthy people. The vast majority of those infected now had no trouble in after life. The bad cases were those who neglected themselves, and were intemperate. The severity of the disease depended more on the soil than on the quality of infection. If restrictive measures were adopted they would have to extend to gonorrhœa, which was responsible for more deaths than syphilis. The contagious element of gonorrhœa was retained longer, and was a prolific cause of kidney disease, salpingitis, metritis, and sterility.

Dissemination of knowledge among our patients should be vigorously prosecuted. Under the domination of the higher emotion of affection sexual intercourse was salutary. Under other circumstances, however, it was injurious and debasing, and much more so was fingering of the genitals and lascivious imaginations. These facts we could impress upon our patients, but public discussion was not advisable.

### ENDOMETRITIS.

Ever since Dr. Polk read his paper on this subject last winter, in which he advised curetting and drainage by means of a gauze packing, considerable interest has been manifested. Dr. W. R. Pryor reintroduced the subject in a paper read at the last meeting of the Academy. He stated that when the discharge was purulent no treatment was so satisfactory as that advocated by Dr. Polk. Intra-uterine applications were liable to prove injurious unless the strictest antiseptic precautions were observed. In this way a simple endometritis might easily be caused to become septic.

Dr. H. T. Hanks said that he had seen many cases cured without a resort to heroic measures. In septic cases the best plan of treatment was certainly curetting, irrigation and packing. This would result in a cure unless the disease had already extended into the abdominal cavity. He had seen good results from electricity, the negative pole being passed into the uterus and the positive being applied to the abdomen by means of the large clay, a felt electrode. As strong a current as the patient could bear should then be applied. Some cases could be cured in this way as quickly as by curetting, but there was no one royal road.

Dr. Bolt thought packing unnecessary in puerperal cases, irrigation, with two to five per cent. carbolic solution being all that was required.

Dr. A. H. Goelet did not think the curette necessary in all cases. He used it only in the fungous variety. Electricity had proved satisfactory in his hands.

Dr. Mundé spoke of the importance of measures directed to improving the general health. Such local treatment as could be given in one's office was waste of time. The os should be dilated by the tupelo tent, and the blunt and sharp curette used; then a fifty per cent. solution of chloride of zinc should be applied. All should be done under antiseptic precautions. Drainage by means of a gauze packing was desirable in some cases. Treatment by electricity prolonged the case, and postponed a cure.

Dr. E. H. Grandin thought electricity useful, except in purulent cases. Then curetting and drainage after washing out with peroxide of hydrogen was the rational treatment to pursue.

Dr. Polk believed that when a septic nidus was left after an incomplete abortion, the large portion of cases recovered with maimed uterine appendages, so that chronic invalidism was the outcome. In such cases, then, he thought the curette should be used freely. This was now the routine treatment with a number of prominent specialists in the city, and was taught to medical students. In chronic endometritis he adopted the same plan of treatment if the condition was at all pronounced, and the more he employed it the better he liked it. The best time for the operation was just before the menstrual period. If the treatment failed the resources of abdominal surgery were still available.

INFANT FEEDING.

Dr. H. Koplik, who has paid considerable attention to this subject in the large children's class at the Good Samaritan Dispensary, made some remarks in reference to sterilized milk at the last meeting of the Section on Pædiatry. He said that among two hundred and twenty-five infants who had continued to use the milk for four or five days or more, one hundred and twenty-five ceased to have any diarrhœal symptoms. Many remained puny, but they were kept alive until the hot weather had passed, and were restored to health in the autumn. The milk used in the dispensary was kept in the sterilizer at 185° F. for forty minutes. Some children did not bear the sterilized milk well, and with these milk boiled and rapidly cooled was resorted to.

All children brought for treatment had used the various infant foods sold in the market, and every one had glycosuria, fifty per cent. of sugar being present in some instances.

In continuing the subject Dr. Jacobi said that no greater injury had been done to the children of the United States than that contributed by the manufacture of infant foods. Even sterilized cow's milk was not a substitute for natural feeding. Many who survived under its uses were invalids for life.

The same subject was under consideration at the last meeting of the county society. Dr. H. D. Chapin read a paper in which he advocated the use of the upper half of a portion of cow's milk allowed to stand in a vessel for several hours. This should be sterilized and diluted with barley-water, which prevented the formation of a curd of too great firmness for digestion.

Dr. J. Lewis Smith thought it advisable to withhold milk entirely for a short time in bad cases. Barley flour, which had been exposed to heat for seven days had part of its starch changed into dextrine, and when mixed with cold water and white of egg, a teaspoonful to a pint made a useful temporary substitute for the milk. Fifteen drops of Forbes' Diastase added to each feeding assisted in the digestive process.

### EXALGINE.

This remedy has been recommended by Dr. C. L. Dana, as unusually successful in chorea. Several cases treated by him who had taken arsenic without benefit, made rapid improvement under exalgine. It was given in capsules in doses of two grains three times daily, after meals, for the first day. On the second two capsules were given three times in the day, and on the third the dose was increased to fifteen grains in the day, at which it was continued.

WM. L. RUSSELL.

151 *East 50th St.*

